# Editorial: Advancements and improvements in general hospital psychiatry

**DOI:** 10.3389/fpsyt.2024.1435498

**Published:** 2024-06-03

**Authors:** Jinya Cao, Ying Zhang, Jing Wei

**Affiliations:** ^1^ Department of Psychological Medicine, Peking Union Medical College Hospital, Chinese Academy of Medical Science and Peking Union Medical College, Beijing, China; ^2^ Department of General Internal Medicine and Psychosomatics, Heidelberg University Hospital Heidelberg, Heidelberg, Germany

**Keywords:** general hospital psychiatry, consultation liaison, organic psychiatric disorder, depression and diabetes, neuropsychiatric SLE (NPSLE), outpatient care

Since the deinstitutionalization of major psychiatric centers, general hospital psychiatry has been playing a more and more important role in providing psychiatric services to the public. But general hospital psychiatry does more than providing services to psychiatric patients in general hospital settings; it also provides consultation-liaison(C-L) services to physical patients with psychiatric comorbidity or psychiatric symptoms due to physical conditions. There is a high prevalence of psychiatric comorbidity in general hospital inpatients of various physical departments. Psychiatric comorbidity results in difficulty in clinical communication, longer hospitalization, worse clinical outcomes, and higher costs. However, psychiatric comorbidities are often neglected and untreated.

Many models of C-L services are designed to best fit tasks such as making diagnoses and prescribing pharmaceuticals, helping with physical patients’ distressing emotions, helping with clinical communications between patients and their doctors, and so on.

In this Research Topic focusing on advancements in general hospital psychiatry, nine research papers from across the globe cover topics including psychiatric service delivery in various general hospital settings(inpatients, emergency, and outpatients), infection prevention strategy in psychiatric patients in general hospitals, pharmaceutical treatment of organic psychiatric disorders (neuropsychiatric systemic lupus erythematosus), and common psychiatric burdens in patients with physical conditions/symptoms(diabetes, postpartum, chest pain, etc.).

## Work methods in different settings

Caspi et al. reported the feasibility of providing home care via online services to suitable psychiatric patients after emergency care with a multi-disciplinary team at Sheba Medical Center, Israel. With the advancement of technologies, patients’ biological status can also be monitored online. This may inspire other clinicians, since medical resources are commonly limited and some patients would prefer home treatment.

In the study of Lundqvist et al., a comprehensive and continuous outpatient service was provided to 373 adult patients registered at 15 psychiatric outpatient clinics in three regions in central and southern Sweden. The outpatient service improved patients’ quality of life via both symptom relief and recovery. Patient-staff relationship was found to have an independent effect on recovery. Simply put, a continuous outpatient service with humane warmth further relieves patients helps them recover.

Casey et al. reviewed medical conditions of 163 patients with primary psychiatric complaints presenting to Ochsner Louisiana State University Shreveport Psychiatric Crisis Unit, USA. In their findings, 50.3% of the patients received interventions prior to medical clearance. Elevated creatine kinase (in 31 patients) was the most common cause for intervention. Additional medical conditions that resulted in medical interventions included tachycardia, elevated serum ethanol level, dehydration, and acute kidney injury. Although the characteristics of patient populations may differ between regions and settings, this study reminds clinicians of the importance of paying attention to medical conditions in general hospital inpatients and emergency patients.

Han et al. reported a bundle management strategy in reducing hospital-acquired pneumonia in hospitalized patients with mental disorders at the mental health center of a tertiary general hospital in Wuhan, China. Infection prevention, isolation of infected patients, environment disinfection, and paying attention to antipsychotics and underlying diseases were applied in combination. The rate of HAP occurrence decreased from 0.95 to 0.52%.

In the study of Dai et al., a questionnaire evaluating hospitalized patients’ expectations for treatment is reported as part of the consultation-liaison effort to help general hospital inpatients and their doctors better communicate treatment expectations in a tertiary general hospital in Beijing, China. Better doctor-patient communication and mutual understanding of medical diagnosis, treatment, and prognosis is integral to shared decision making, increases patient’s compliance to treatment, and improves clinical outcome.

## Studies of psychiatric disorders in physical conditions

One general rule is that the relationship between psychiatric disorders and physical conditions are bi-directional. But it is always difficult to tell how and to what extent one influences another. Specific diseases/conditions merit specific research.

Geng et al. reviewed the records of 160 inpatients with systemic lupus erythematosus (SLE) who required psychiatric consultation for further therapeutic intervention in a tertiary general hospital in Beijing, China. In these patients, 86.3% met the diagnostic criteria of at least one mental disorder, the most common being delirium. Patients with delirium have the highest mortality rate in patient groups. Antipsychotic usage was found to decrease death risk for patients with neuropsychiatric systemic lupus erythematosus (NPSLE). This stresses the importance of early recognition and treatment of psychiatric symptoms in SLE patients.

In the study of Sun et al., postpartum mental disorders were diagnosed in a sample of 284 parturients in a tertiary general hospital in Beijing, China. The risk of postpartum depression, anxiety disorders, and obsessive-compulsive disorder was 9.125 times, 7.310 times, and 6.259 times higher in postpartum women with high psychological stress levels related to delivery than in those with low psychological stress levels respectively. Future interventions focusing on psychological stress related to delivery could be very valuable in postpartum mental disorder prevention.

Both diabetes and depression are very common and cause a significant global health burden. A lot of research into their comorbidity have been done. In the study of Kim et al., the relationship between type 2 diabetes and depression was reconsidered using the National Health Insurance Sharing Service (NHISS) database of the National Health Insurance Service (NHIS) of South Korea. In contrast to the common impression that depression can increase the risk of type 2 diabetes, in this study it was found that depression and antidepressant medications were not contributory factors for type 2 diabetes after adjusting for other physical comorbidities.

The study of Zarean et al. Investigated depression and anxiety in healthy controls, patients with cardiac chest pain, and patients with non-cardiac chest pain in multiple medical centers across Shahrekord, Iran. Patients with non-cardiac chest pain had higher levels of depression and anxiety and lower quality of life than patients with cardiac chest pain and healthy controls.

In summary, this edition includes several new studies on setting specific work methods and disease/condition-specific psychiatric comorbidity in general hospital psychiatry. But these are only a some of the wide topics that need to be investigated in the field. We look forward to more nuanced studies into methodological and scientific aspects of general hospital psychiatry in the future.

## Author contributions

JC: Writing – original draft. YZ: Writing – original draft. JW: Writing – review & editing.

